# Herb-Induced Liver Injury Related to *Reynoutria multiflora* (Thunb.) Moldenke: Risk Factors, Molecular and Mechanistic Specifics

**DOI:** 10.3389/fphar.2021.738577

**Published:** 2021-09-02

**Authors:** Xing-Ran Zhai, Zheng-Sheng Zou, Jia-Bo Wang, Xiao-He Xiao

**Affiliations:** ^1^ Peking University 302 Clinical Medical School, Beijing, China; ^2^ Medical School of Chinese PLA, Beijing, China; ^3^ Senior Department of Hepatology, the Fifth Medical Center of PLA General Hospital, Beijing, China; ^4^ School of Traditional Chinese Medicine, Capital Medical University, Beijing, China; ^5^ China Military Institute of Chinese Medicine, the Fifth Medical Center, Chinese PLA General Hospital, Beijing, China

**Keywords:** drug-induced liver injury, herb-induced liver injury, predisposing factors, molecular mechanisms, *Polygonum multiflorum* Thunb., *Reynoutria multiflora* (Thunb.) Moldenke

## Abstract

Herbal medicine is widely used in Asia as well as the west. Hepatotoxicity is one of the most severe side effects of herbal medicine which is an increasing concern around the world. *Reynoutria multiflora* (Thunb.) Moldenke (*Polygonum multiflorum* Thunb., PM) is the most common herb that can cause herb-induced liver injury (HILI). The recent scientific and technological advancements in clinical and basic research are paving the way for a better understanding of the molecular aspects of PM-related HILI (PM-HILI). This review provides an updated overview of the clinical characteristics, predisposing factors, hepatotoxic components, and molecular mechanisms of PM-HILI. It can also aid in a better understanding of HILI and help in further research on the same.

## Introduction

Herbal medicine, as a complementary and alternative medicine, is widely used in Asia as well as the west. Herbal medicine is generally considered to be natural, green, and harmless. In reality, herbal medicine may also have side effects, such as hepatotoxicity, nephrotoxicity, cardiotoxicity, neurotoxicity, and carcinogenicity. Hepatotoxicity is the most common side effect of great concern. The hepatotoxicity of drugs can be intrinsic or idiosyncratic (European Association for the Study of the Liver, 2019). The former refers to a liver injury that is closely related to drug dosage and timing, can be predicted, and has insignificant individual differences; the latter refers to a liver injury that is not correlated with the drug dosage and timing, is unpredictable, seen in a few people, and has a significant individual difference.

Herbal medicine is a common cause of drug-induced liver injury (DILI). A recent study carried out in Asian countries focusing on the causes of DILI reported that herbal medicine accounts for about 25% of all DILI (Byeon et al., 2019; Shen et al., 2019). However, very few studies focus on herb-induced liver injury (HILI), and little is known about species, components, mechanisms, and predisposing factors of herbs that cause liver injury. Common herbs that can cause HILI include *Reynoutria multiflora* (Thunb.) Moldenke (*Polygonum multiflorum* Thunb., PM), *Cullen corylifolium* (L.) Medik., *Corydalis yanhusuo* (Y.H.Chou and Chun C. Hsu) W.T.Wang ex Z.Y.Su and C.Y.Wu, etc., of which the most common is PM, accounting for 32.3% of all cases (Byeon et al., 2019). PM-related HILI (PM-HILI) is seen only in a few of the patients consuming this herb.

PM, the dried tuberous root of *Reynoutria multiflora* (Thunb.) Moldenke, when used as a traditional tonic and dietary supplement is believed to possess hypolipidemic, anti-atherogenic, anti-aging, immunity-enhancing, neuroregulatory, hepatoprotective, and anti-inflammatory properties. These potentially beneficial effects contradict their role in liver injury. However, the potential beneficial effects and rare liver injury are common features of many herbs that cause liver injury. An updated review can help in better understanding the predisposing factors, hepatotoxic components, and molecular mechanisms of PM-HILI, and aid in its diagnosis, treatment, and prevention. It can also aid in a better understanding of HILI and help in further research on the same.

Although *Polygonum multiflorum* Thunb. is not an international standard Latin name, but it is widely used in scientific literature. In order to make it easier for readers to understand and relate to other literature, the abbreviation PM is used in this review.

## Clinical Characteristics of PM-HILI

Reports of PM-HILI from China and other countries (But et al., 1996; Park et al., 2001; Mazzanti et al., 2004; Panis et al., 2005; Jung et al., 2011; Lian et al., 2013; Zhang Y. et al., 2013; Dong H. et al., 2014; Zhu et al., 2015; Luo et al., 2016; Li CY. et al., 2017; Han Z. X. et al., 2019; Fu et al., 2020) have increased in recent years (Table 1). Compared to other DILI, PM-HILI does not present specific clinical and pathological features. Hepatocellular injury is very common and cholestasis and mixed liver injury are rare. Common manifestations are jaundice and markedly increased levels of biochemical parameters such as total bilirubin, alanine aminotransferase, aspartate aminotransferase, etc. (Park et al., 2001; Mazzanti et al., 2004; Panis et al., 2005; Jung et al., 2011; Dong H. et al., 2014; Zhu et al., 2015; Li H. et al., 2017; Wang Y. et al., 2019). Most patients with PM-HILI have a good prognosis, although some patients may present with acute liver failure, chronic liver injury, or even death. PM-HILI with underlying chronic liver disease, particularly alcoholic liver disease, has a worse prognosis and is more likely to develop into chronic DILI (Jing et al., 2019). Studies suggest that PM-HILI is an idiosyncratic drug-induced liver injury (IDILI). Clinical epidemiological analysis has found that PM-HILI only occurs in a very small number of individuals taking PM, which is a low-probability event (Zhang et al., 2020b). PM is usually prescribed at a wide range of doses over large periods in patients, which suggests the lack of a clear dose-dependent relationship (Zhu et al., 2015; Tu et al., 2019a; Zhang et al., 2020b). In clinical practice, PM has been prescribed at a dosage within the recommended range of Chinese Pharmacopeia (3–6 g/day for raw PM, 6–12 g/day for processed PM) in patients with PM-HILI (Lei et al., 2015; Li CY. et al., 2017). PM-HILI is most commonly seen in patients with immune-related diseases such as vitiligo, psoriasis, rheumatoid arthritis, seborrheic alopecia, which are often accompanied by immune stress or mild elevation of inflammatory cytokines (Tu et al., 2019a), suggesting that immune stress may be an important predisposing factor for PM-HILI. Experimental animal studies have shown that only a long-term and large-dose administration of PM can exert hepatotoxic effects in healthy animals, while the corresponding dose is clinically difficult to achieve (Li et al., 2013; Zhu et al., 2014; Wang T. et al., 2015; Yang M. et al., 2016). However, PM can cause liver injury when administrated at a routine clinical dose within a short period in susceptible animal models (Li et al., 2015; Xie et al., 2016). Nevertheless, the phenomenon of high-dose and long-term administration of PM is also observed in clinical practice, and it cannot be ruled out that PM also exerts direct hepatotoxic effects when administered at very high doses.

**TABLE 1:** Representative studies^a^ on liver injury caused by PM.

Number of cases	Duration	Male: Female	Age, year	Duration of intake, day	PM type	Type of liver injury (%)	Number of deaths (%)	References
36	2000–2012	23: 13	24–73	<7 (13.9%); 7–14 (11.1%); 14–30 (30.6%); 30–90 (30.6%); >90 (11.1%); Unknown (2.8%)	RPM and PPM	Hepatocellular (58.3), Cholestasis (5.6), mixed (36.1)	2 (5.6)	Zhang et al. (2013) (Zhang et al., 2013b)
52	2006–2012	30: 22	22–69	5–120	PPM	Hepatocellular (57.7), Cholestasis (17.3), mixed (25.0)	0	Lian et al. (2013) (Lian et al., 2013)
66	2009–2014	35: 31	8–63	1–365	RPM and PPM	Hepatocellular (92.4), Cholestasis (1.5), mixed (6.1)	1 (1.5)	Zhu et al. (2015) (Zhu et al., 2015)
60	2006–2010	28: 32	43.5^b^	11–168	RPM	Hepatocellular (60.0), Cholestasis (15.0), mixed (25.0)	1 (1.7)	Luo et al. (2016) (Luo et al., 2016)
133	2012–2016	not reported	8–77	5–224	RPM and PPM	not reported	not reported	Han et al. (2019) (Han et al., 2019b)
140	2009–2018	60: 80	19–74	1–150	RPM and PPM	Hepatocellular (94.3), Cholestasis (2.9), mixed (2.9)	1 (0.71)	Fu et al. (2020) (Fu et al., 2020)

Abbreviations PM, Reynoutria multiflora (Thunb.) Moldenke; RPM, raw Reynoutria multiflora (Thunb.) Moldenke; PPM, processed Reynoutria multiflora (Thunb.) Moldenke.

a Studies with more than 30 cases are listed.

b Average age.

## Predisposing Factors and Biomarkers

### Mild Immune Stress

Clinical analysis of PM-HILI cases has shown immune stress as one of the important predisposing factors for PM-HILI (Tu et al., 2019a). Tu et al. discovered that high-dose (50 g/kg) and long-term administration of PM to healthy rats did not cause obvious liver injury (Tu et al., 2015), but administration of PM with 2 times clinical-equivalent dose (1.08 g/kg) to the MIS model rats induced by lipopolysaccharide (LPS) led to obvious liver injury, by which a model for evaluating the idiosyncratic hepatotoxicity of PM based on immune stress was established for the first time (Li et al., 2015). LPS is a major component of the cell wall of Gram-negative bacteria, which can activate the immune system and promote the release of pro-inflammatory cytokines, such as tumor necrosis factor (TNF)-α, interleukin (IL)-1β, IL-6, etc. A low dose of immune activator (such as LPS) can induce MIS, a mild and damage-free inflammatory response that stimulates immune stress or aberrant activation of immune cells of the body, thus increasing the susceptibility to PM-HILI.

Using multifactor detection, metabolomics approach, and receiver operating characteristic curve analysis, Tu et al. (Tu et al., 2019b) identified various susceptibility biomarkers for PM-HILI with MIS idiosyncratic hepatotoxic rat model, including 12 plasma chemokines or cytokines (IL-1β, IL-6, IL-10, interferon (IFN)-γ, IFN-γ induced protein (IP)-10, macrophage chemoattractant protein (MCP)-1, MIP-1α, MCP-3, growth-related oncogene protein-α, granulocyte-macrophage colony-stimulating factor, TNF-α, Rantes) and 9 metabolites (L-Phenylalanine, Creatinine, L-Glutamine, N1, N5, N10-Tricoumaroyl spermidine, Dopamine, Acetone cyanohydrin, Glycocholic acid, Deethylatrazine, L-Valine).

A study that examined susceptibility biomarkers for PM-HILI conducted by Zhang et al. (Zhang et al., 2020b) discovered significant differences in serum metabolites between susceptible and tolerant individuals. A total of 25 different metabolites were screened, involved in glycerol phospholipid, sphingolipids, fatty acid, histidine, and aromatic amino acid metabolism. A significant proportion of these metabolites are directly or indirectly involved in the regulation of immune and inflammatory responses. The serum levels of TNF-α, IL-1β, and IL-6 were significantly increased in patients susceptible to PM-HILI compared with those tolerant to PM-HILI, with a threefold increase in TNF-α, and its levels significantly correlated with multiple differential metabolites. It is recently reported that serum cytokine TNF-α and chemokine CCL2 or vascular endothelial growth factor (VEGF) as potential biomarkers of PM-HILI (Tu et al., 2021).

In short, when plasma and liver concentrations of inflammatory cytokines such as TNF-α, IL-1β, IL-6, MCP-1, VEGF are elevated, PM should be used with caution.

### Gene Polymorphism

#### Immune-Related Gene Polymorphism

In the last decade, numerous genome-wide association studies have linked human leukocyte antigens (HLAs) to IDILI susceptibility (Kaliyaperumal et al., 2018). Li et al. (Li et al., 2019) discovered that the frequency of HLA-B*35:01 allele in patients with PM-HILI is approximately 45.4%, significantly higher than in the general Han Chinese population (2.7%). The incidence of PM-HILI in HLA-B*35:01 allele carriers is eight times higher than that in non-carriers. Therefore, it has been hypothesized that the HLA-B*35:01 allele is one of the susceptibility genes of PM-HILI in the Han Chinese population, and has potential value in predicting the occurrence of PM-HILI. A study by Yang et al. (Yang et al., 2020) showed the frequency of the HLA-B*35:01 allele is 41.1% in patients with PM-HILI, which is consistent with the above findings and confirms the association between HLA-B*35:01 allele and PM-HILI. The relationship between single-nucleotide polymorphisms (SNPs) and PM-HILI was investigated and rs1055348 in the HLA-B gene was identified as a potential susceptibility SNP specific to PM-HILI. Rs1055348 has a high association with the HLA-B*35:01 allele and may serve as a tag for the HLA-B*35:01 allele. Rs1055348 can be an alternative to HLA-B*35:01 allele due to its low cost and easy detection. The above studies identified potential genetic susceptibility markers for PM-HILI, suggesting that screening for HLA-B*35:01 allele or tag SNP rs1055348 may be beneficial in the identification of susceptible populations of PM-HILI in the Han Chinese population. It remains to be investigated whether HLA-B*35:01 or rs1055348 has predictive value for susceptible individuals in non-Han Chinese populations.

#### Metabolic Enzyme-Related Gene Polymorphism

Cytochrome P-450 (CYP450) has polymorphisms. Alterations in allele frequency may lead to an increase or decrease in CYP450 enzyme activity, which is one of the reasons for individual differences in drug metabolism and drug response. Ma et al. (Ma et al., 2014) found that the frequency of CYP1A2*1C allele in patients with PM-HILI is 46.5%, higher than that in healthy controls (27.9%), suggesting CYP1A2*1C mutation may cause PM-HILI. CYP1A2 is an important enzyme involved in the biotransformation of emodin (Ma et al., 2014). CYP1A2*1C mutation is associated with decreased CYP1A2 activity (Zhou et al., 2009). The metabolism of emodin, an anthraquinone derivative in PM, may be inhibited, causing accumulation of emodin and promoting the progression of liver injury. It should be noted that the content of emodin in PM is low, and it may be difficult to reach a dose concentration clinically that can cause liver injury. In contrast, consumption of herbs rich in emodin, such as rhubarb, causes much lesser liver injury than PM-HILI clinically. Currently, no animal and cell studies have confirmed the role of the CYP1A2*1C allele in the occurrence of PM-HILI.

### Bile Acids

A targeted metabolomics study by Dong et al. (Dong et al., 2015) showed that bile glycodeoxycholic acid levels and serum hyodeoxycholic acid (HDCA) levels are significantly decreased in PM-HILI rats, which may serve as potential biomarkers for PM-HILI. A study conducted by Zhao et al. (Zhao et al., 2017) showed that serum HDCA can be a potential biomarker for PM-HILI, and the result showed significantly increased serum HDCA in PM-HILI rats, in contradiction to the previous study. This may be due to the different dosages of PM administration and the different time points of HDCA measurement. A significant rise in Tauro-β-muricholic acid levels in urine was observed, which may also be a potential biomarker for PM-HILI. These two biomarkers are positively correlated with the dosage and timing of PM and revert to the normal levels during the recovery period, suggesting good sensitivity. Exploring the sensitivity and specificity of biomarkers for PM-HILI is beneficial for accurate diagnosis and prompt commencement of therapy.

### MicroRNA-122

It has been proposed that microRNA-122, a hepatocyte-specific microRNA, is an ideal potential biomarker for liver disease (Fontana, 2014). Fan et al. (Fan et al., 2015) showed that serum microRNA-122 may also be a potential biomarker for PM-HILI. However, microRNA-122 may not be specific to PM-HILI, and its efficacy in clinical practice needs further testing.

## Idiosyncratic Hepatotoxicity-Related Components in PM

The identification of hepatotoxic components is important to reveal the hepatotoxic effect of herbal medicine. The chemical composition of PM is complex and includes stilbene glycosides, anthraquinones, tannins, flavonoids, phospholipids, micronutrients, etc. The current focus of literature reports has been on the hepatotoxic effects of stilbene glycosides and anthraquinones (Lin et al., 2015; Li et al., 2016; Zhang et al., 2017a; Lin et al., 2017; Zhang et al., 2018; Han L. et al., 2019; Lin Y. et al., 2019; He et al., 2019; Li H.-Y. et al., 2020; Zhang et al., 2020a; Li RL. et al., 2020; Liu et al., 2020).

**FIGURE 1:**
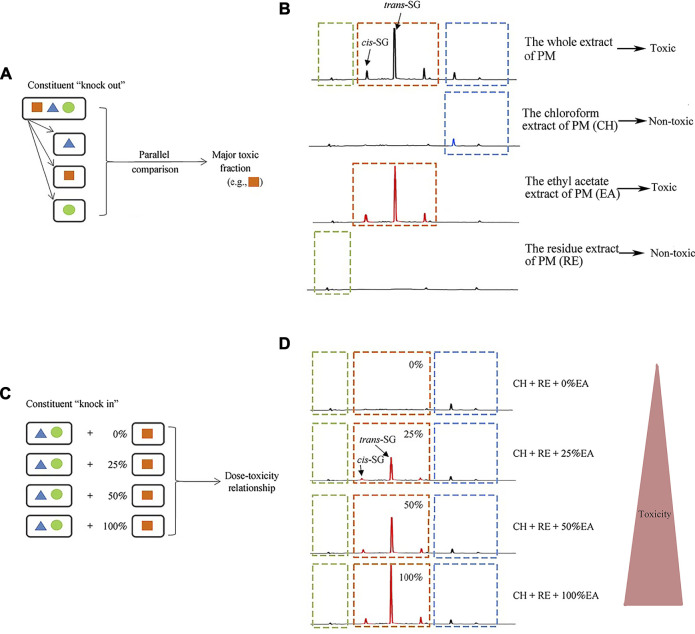
Administration of the stilbene-containing ethyl acetate extract of PM to LPS-treated rats resulted in hepatotoxicity. Adapted from reference (Li C. et al., 2017), with permission from Springer, 2021. Abbreviations: *trans*-SG, *trans*-2,3,5,4'-tetrahydroxystilbene-2-O-β- glucoside; *cis*-SG, *cis*-2,3,5,4'-tetrahydroxystilbene-2-O-β-glucoside; PM, *Reynoutria multiflora* (Thunb.) Moldenke; CH, the chloroform extract of PM; EA, the ethyl acetate extract of PM; RE, the residue extract of PM.

**FIGURE 2:**
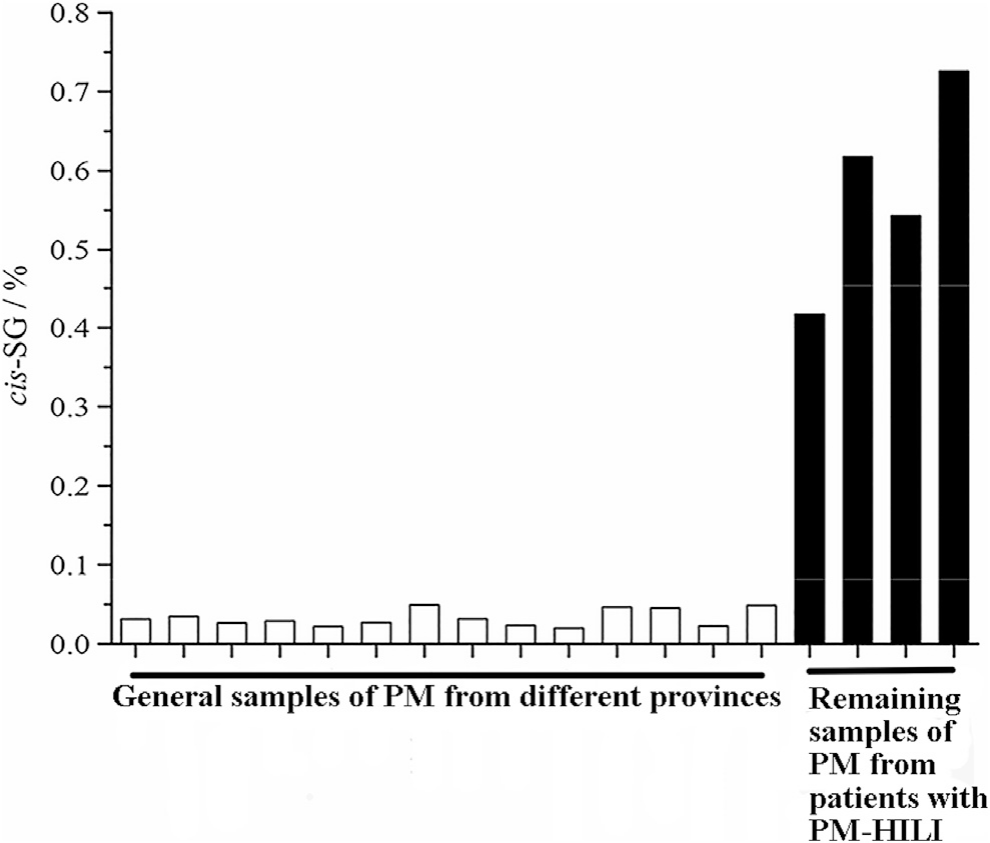
The content of *cis*-SG in the remaining samples of PM from patients with PM-HILI is significantly higher than that of the general samples of PM from different provinces. Adapted with permission from reference (Zhang et al., 2017a). Abbreviations: *cis*-SG, *cis*-2,3,5,4'-tetrahydroxystilbene-2-O-β-glucoside; PM, *Reynoutria multiflora* (Thunb.) Moldenke; PM-HILI, PM-related herb-induced liver injury.

Li et al. (Li C. et al., 2017) found that ethyl acetate (EA) extract is responsible for the idiosyncratic hepatotoxicity of PM using the "knock-out" and "knock-in" strategies (Figure 1). Using mass spectrometry, it has been identified that the EA extract of PM consists of *trans*-2,3,5,4'-tetrahydroxystilbene-2-O-β-glucoside (*trans*-SG), *cis*-2,3,5,4'-tetrahydroxystilbene-2-O-β-glucoside (*cis*-SG), and emodin-8-O-glucoside (EG). Li et al. (Li C. et al., 2017) used monomers and confirmed that *cis*-SG, and not *trans*-SG, is the important component responsible for the idiosyncratic hepatotoxicity in PM. *Cis*-SG shows a definite dose-toxicity relationship in the MIS model and the dose is nearly equivalent to the clinical dosage by weight. The content of *cis*-SG in remaining samples of PM from patients with PM-HILI is significantly higher than that of general samples of PM from different provinces further proves that *cis*-SG is a hepatotoxicity-related component of PM-HILI (Figure 2) (Zhang et al., 2017a).

**FIGURE 3:**
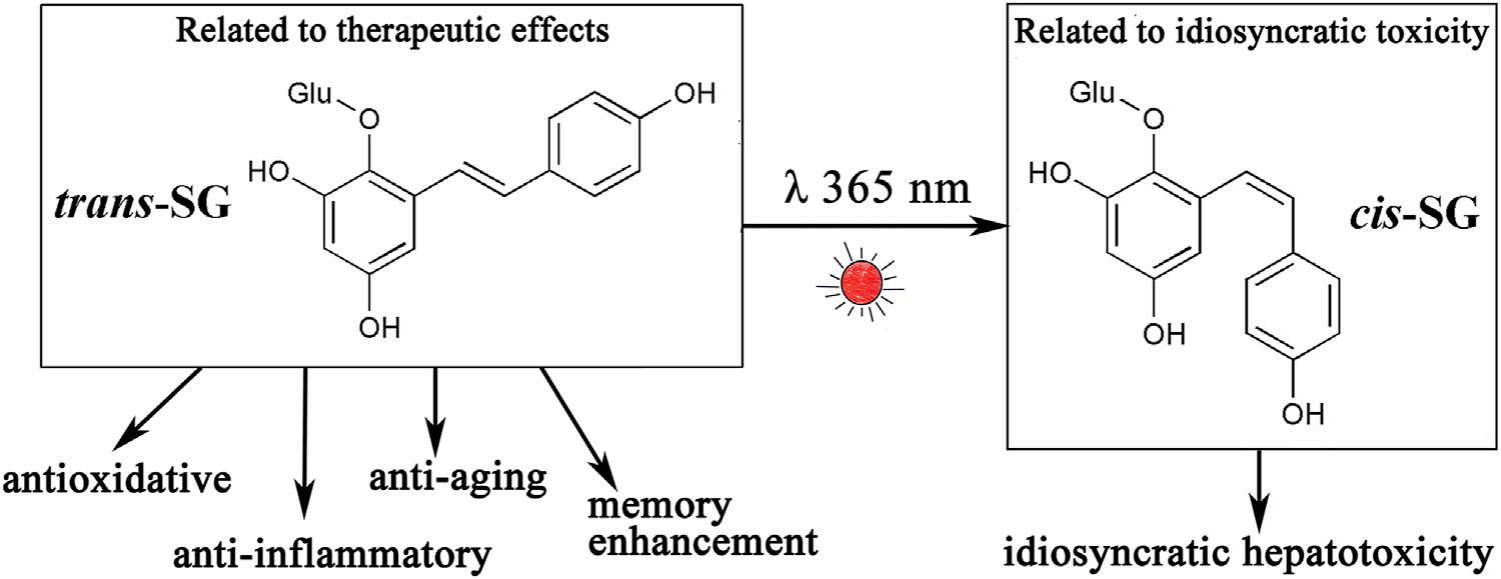
*Trans*-SG can be transformed into its *cis*-isomer (*cis*-SG) by ultraviolet light or sunlight. *Trans*-SG is a component responsible for therapeutic effects, however, *cis*-SG is an idiosyncratic hepatotoxic component. Abbreviations: *trans*-SG, *trans*-2,3,5,4'-tetrahydroxystilbene-2-O-β- glucoside; *cis*-SG, *cis*-2,3,5,4'-tetrahydroxystilbene-2-O-β-glucoside.

The naturally occurring component in PM is largely *trans*-SG, and the content of *cis*-SG in PM is low. *Trans*-SG is the component responsible for beneficial effects and has properties of anti-oxidation (Tao et al., 2011; Zhang et al., 2012), anti-inflammation (Zhang F. et al., 2013; Huang et al., 2013), anti-senescence (Han et al., 2012; Büchter et al., 2015), and enhancing memory (Wang et al., 2011; Chen et al., 2016). Zhang et al. (Zhang et al., 2017a) studied the relationship between levels of *cis*-SG and idiosyncratic liver injury of PM and found that the higher the *cis*-SG content, the greater the hepatotoxicity. They also showed that PM does not cause liver injury on the MIS model when the *cis*-SG content is less than 0.10%, suggesting an upper safety limit for quality control of *cis*-SG in PM. Therefore, c*is*-SG is the component responsible for idiosyncratic hepatotoxicity in PM. However, ultraviolet irradiation can induce the transformation of *trans*-SG to *cis*-SG (Dong L. H. et al., 2014), resulting in the transformation of effective components into toxic components (Figure 3) and increasing the risk of liver injury. Therefore, exposure to light should be avoided during the preparation and storage of PM.

Zhang et al. (Zhang et al., 2020a) studied EG in the EA extract of PM and found that EG causes liver injury in the MIS rat model in a dose-dependent manner and the dose is nearly equivalent to the clinical dose by weight. It is indicated that EG is an important component in PM that is responsible for idiosyncratic hepatotoxicity. The upper safety limit of quality control of EG in PM is 0.17%, determined using the method described earlier by Zhang et al. (China Association of Chinese Medicine, 2020).

Equimolar amounts of emodin do not cause liver injury, and the toxicity of combined anthraquinone is significantly higher than free anthraquinone in idiosyncratic hepatotoxicity, in contrast with the results of direct hepatotoxicity caused by the two compounds at very large doses (Yang M. et al., 2016). This helps in better understanding the fact that the toxicity of PM decreases after processing.

**FIGURE 4:**
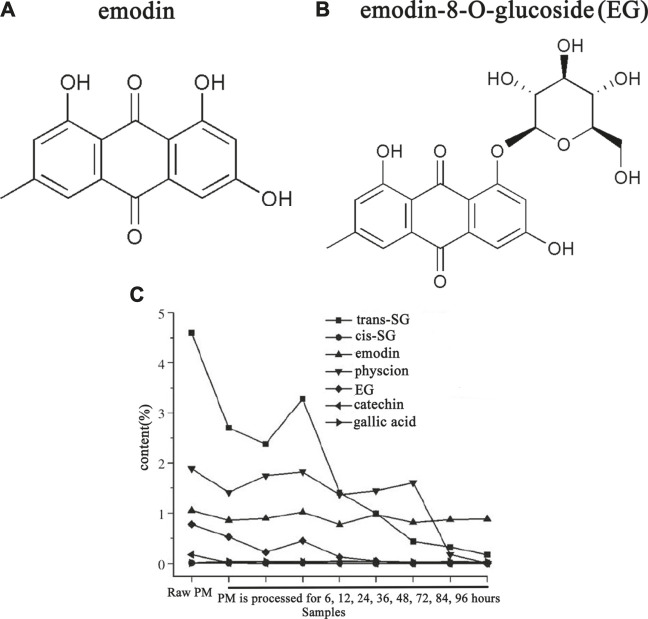
The content of emodin-8-O-glucose (EG) of PM was reduced after processing. Adapted with permission from reference (Gao et al., 2017). Abbreviations: *trans*-SG, *trans*-2,3,5,4'-tetrahydroxystilbene-2-O-β-glucoside; *cis*-SG, *cis*-2,3,5,4'- tetrahydroxystilbene-2-O-β-glucoside; EG, emodin-8-O-glucoside; PM, *Reynoutria multiflora* (Thunb.) Moldenke.

The finding of Ma et al. (Ma et al., 2015) and Gao et al. (Gao et al., 2017) showed that both the toxicity as well as the content of EG is reduced after processing (Figure 4), which indicates the hepatotoxicity of PM is highly correlated with EG. Reducing the idiosyncratic hepatotoxic components (or the susceptible components) of PM by processing is one of the important approaches to reduce toxicity.

Anthraquinones such as emodin (Lin et al., 2015; Zhang et al., 2018; Li H.-Y. et al., 2020), rhein (Li H.-Y. et al., 2020), and other components such as torachrysone-O-hexose (Han L. et al., 2019), dianthrones (Li H.-Y. et al., 2020), polygonnodes C1-C4 (Yang JB. et al., 2016), catechin (Li et al., 2016), etc. with intrinsic hepatotoxicity have also been investigated. Although the above components cause liver injury when administrated in large quantities, the quantity of the above components is very low in the clinical dosage of PM. Therefore, PM-HILI cannot be independently caused by the above components. However, they may play a secondary or synergistic role in the occurrence of PM-HILI. Recognition of this may contribute to a more complete understanding of the complex multi-component mechanisms of PM-HILI.

## Molecular Mechanisms

Current studies have shown that PM-HILI is considered to be IDILI and only occurs in a small number of susceptible individuals. However, in addition to IDILI, direct hepatotoxicity may be seen in specific PM-HILI patients who received a large dosage of PM. PM-HILI may be a result of a synergistic effect of multiple components and multiple mechanisms. Studies have shown that the molecular mechanism of PM-HILI is related to the inflammatory reaction, alterations of drug-metabolizing enzymes, apoptosis, and disorders of bile acid metabolism. A summary of the related mechanisms of PM-HILI around the two main susceptible components (*cis*-SG and EG) is presented below.

### Inflammatory Reaction

Peroxisome proliferator-activated receptors (PPARs) belong to the nuclear receptor transcription factor superfamily. PPAR-γ, one subtype of PPARs, plays an important role in immune response and has a role in inhibiting the expression of inflammatory cytokines (Wu et al., 2020). Meng et al. (Meng et al., 2017) found that the mechanism of *cis*-SG in PM inducing idiosyncratic liver injury in the MIS rat model is associated with down-regulated expression of PPAR-γ, activation of the nuclear factor-κB signal pathway, secretion of proinflammatory cytokines such as TNF-α and IL-6 by monocytes/macrophages; and the induction of hepatocyte apoptosis. Thus, the administration of a PPAR-γ agonist (such as pioglitazone) in advance may prevent liver injury caused by *cis*-SG. Studies by Zhang et al. (Zhang et al., 2020a) and He et al. (He et al., 2017) have shown *trans*-SG in PM synergistically enhances the liver injury caused by LPS/EG and LPS/*cis*-SG and is associated with increased expression of proinflammatory cytokines and enhancement of inflammatory activity. Pan et al. (Pan et al., 2020) found that the activation of inflammasomes may also be a mechanism by which *cis*-SG induces IDILI. Previous studies have shown that MIS is a risk factor for PM-HILI. The pro-inflammatory effects of *cis*-SG and *trans*-SG and the involvement of inflammasomes prove that inflammation plays a role in PM-HILI.

### Alterations of Drug-Metabolizing Enzymes

The up or down-regulation of drug-metabolizing enzymes may affect the metabolism of drugs and slow down the elimination of drugs from the body, and lead to toxic side effects. In recent years, studies have found that the chemical composition of PM affects the activity of drug-metabolizing enzymes.

Studies (Wang et al., 2015a; Wang M. X. et al., 2016) have found that emodin, an anthraquinone in PM, has a role in inducing many CYP450 enzymes, such as CYP1A1, CYP1B1, CYP3A, CYP1A, CYP2B, CYP2E1, etc. *Trans*-SG has been found to increase the expression of CYP1A2 and CYP3A4 both in mice and in human normal liver L02 hepatocytes, and thus exacerbates the hepatotoxicity induced by acetaminophen in mice (Xu et al., 2017). Another study (Xing et al., 2019) also shows that *trans*-SG induces the activity of CYP1A2, and thus enhances the metabolism of emodin. Several components in PM have inhibitory effects on several uridine diphosphate glucuronosyltransferases (UGTs). EG, emodin-8-O-beta-D-glucoside, hydroxy emodin, emodin, *cis*-emodin dianthrones, *trans*-emodin dianthrones, and polygonumnolide C2 have a strong inhibitory effect on UGT1A1 (Wang Q. et al., 2016; Wang et al., 2017; Wang et al., 2018; Wang Q. et al., 2019); emodin plays a role in down-regulating the gene and protein expression of UGT2B7 (Wu et al., 2018); *trans*-SG has a role in down-regulating UGT1A8 levels (Ma et al., 2013). The effect of the above components on metabolic enzymes may be involved in the mechanisms of PM-HILI in susceptible individuals. However, no animal studies have demonstrated that alteration or knock-out of metabolic enzymes independently affects PM-HILI.

### Apoptosis

Components related to toxicity in PM can promote the production of reactive oxygen species (ROS) and induce cell apoptosis, which may be the common endpoint of many mechanisms. A proteomic study (Lin L. et al., 2019) showed that emodin affects the oxidative phosphorylation pathway by inhibiting the function of mitochondrial respiratory chain complexes, which leads to an increase of ROS, the decrease of mitochondrial membrane potential, dysfunction of ATP synthesis, an increase in cytochrome C (Cyt C) release into the matrix, and the activation of the apoptotic factor aspartic-specific caspase leading to mitochondrial energy metabolism disorder and hepatocyte apoptosis. Another study (Zhang et al., 2017b) found that the process by which emodin induces apoptosis may be mitochondrial cyclophilin D. Lai et al. (Lai et al., 2009) found that the ataxia-telangiectasia mutation (ATM) gene lies downstream of ROS and upstream of p53. Emodin leads to mitochondrial-dependent apoptosis by activating the ROS-ATM-p53-Bax signaling pathway. Rhein was also reported (Bounda et al., 2015) to induce cell apoptosis via the mitochondrial pathway, and the specific mechanism is similar to the abovementioned process. Due to the low content of emodin in PM, it may be difficult to obtain a hepatotoxic dosage causing liver injury clinically. However, the mild mitochondrial damage caused by low-dose emodin may result in the release of damage-associated molecular patterns, which may be concurrently involved in the pathogenesis of PM-HILI with other contributing risk factors.

### Disorders of Bile Acid Metabolism

PM-HILI can be pathologically described as acute hepatitis, followed by cholestatic hepatitis (Wang Y. et al., 2019). Studies have shown that PM can cause bile acid metabolism disorders, and the mechanism may involve proteins and enzymes that affect the metabolism and transport of bile acid. Kang et al. (Kang et al., 2017) used sandwich-cultured rat hepatocytes to study the effects of anthraquinones such as emodin, chrysophanol, and physcion in PM on bile acid. Results showed that all three anthraquinones have a role in causing intracellular cholestasis. Of these, emodin inhibited the bile salt export pump (Bsep); chrysophanol inhibited the multidrug resistance-associated protein 2, and all three anthraquinones inhibited the basolateral efflux transporters. Xue et al. (Wang X. et al., 2019) found that emodin inhibits Bsep by interfering with the interaction of 5’ adenosine monophosphate-activated protein kinase and farnesoid X receptor (Fxr) in the liver, thereby playing a role in promoting intrahepatic cholestasis. Sun et al. (Sun et al., 2021) studied the effect of SG on bile acid and found significantly increased serum bile acid levels. SG inhibited Fxr and Bsep, leading to bile acid accumulation. SG inhibited 25-hydroxycholesterol-7alpha-hydroxylase (CYP7B1) and interfered with the alternative pathway of bile acid synthesis.

Intrahepatic bile acid accumulation may have toxic effects on hepatocytes. On the one hand, bile acid disrupts the integrity of the mitochondrial membrane through its washing effect on lipids, leading to the production of ROS and release of Cyt C, resulting in the oxidative modification of lipids, proteins, and nucleic acids, and ultimately mitochondrial-dependent apoptosis (Perez and Briz, 2009). On the other hand, bile acid causes endoplasmic reticulum stress and activates the programmed cell death pathway (Maillette de Buy Wenniger and Beuers, 2010). A causal relationship between increased bile acids and the occurrence of PM-HILI is unclear, but it cannot be ruled out that intrahepatic cholestasis may be a risk factor for PM-HILI.

### Others

Emodin-glutathione (GSH) adducts (Qin et al., 2016; Jiang et al., 2017) can be formed by emodin or its metabolites of PM, and consequently, lead to hepatotoxicity. Emodin (Qin et al., 2016; Jiang et al., 2017) and aloe-emodin (Dong et al., 2017) have been reported to have a role in reducing GSH, an important intracellular antioxidant. GSH plays a role in maintaining the normal redox balance and protects the liver from oxidative stress. Reduction of GSH may lead to decreased ability to scavenge oxygen free radicals, resulting in oxidative stress response (Chen et al., 2009). However, the amounts of anthraquinones in PM may be too small to deplete GSH and cause liver injury, as the daily dose of even acetaminophen should be more than 4 g for GSH depletion to take place.

## Hypotheses of the Mechanism of PM-HILI

Several hypotheses about the mechanism of IDILI have been established (Roth and Ganey, 2011; Bai et al., 2017), including inflammatory stress hypothesis, genetic polymorphism hypothesis, hapten hypothesis, mitochondrial dysfunction hypothesis, etc. Inflammatory stress hypothesis refers to that a state of inflammation stress of the body may increase the susceptibility of the liver to some drugs, resulting in IDILI. Several drugs of idiosyncratic nature like diclofenac (Deng et al., 2006), ranitidine (Luyendyk et al., 2003), chlorpromazine (Buchweitz et al., 2002), trovafloxacin (Shaw et al., 2007), etc. have been proven to induce IDILI in LPS models. HLAs- and metabolic enzyme-related gene polymorphism has been closely associated with IDILI. Studies have shown that HLA-B*57:01 (Monshi et al., 2013), HLA-A*33:03 (Hirata et al., 2008), HLA-DQA1*02:01 (Spraggs et al., 2011), etc. are related to IDILI caused by flucloxacillin, ticlopidine, lapatinib, respectively. The gene polymorphism of UGT2B7, CYP2C8 (Daly et al., 2007), and CYP2E1 (Vuilleumier et al., 2006) has been reported to be related to IDILI caused by isoniazid. Hapten hypothesis refers to that some reactive drug metabolites bind to cellular proteins and act as haptens, which may induce an adaptive immune response (Uetrecht, 2007) and damage tissue by activating cells to release ROS, proteases, cytokines, etc. Studies have found that IDILI induced by multiple drugs including halothane (Bird and Williams, 1989), sulfamethoxazole (Callan et al., 2009), piperacillin (El-Ghaiesh et al., 2012), etc. is related to the hapten hypothesis. Multiple drugs (troglitazone, diclofenac, nimesulide, etc.) associated with IDILI exhibit a clear mitochondrial hazard (Boelsterli and Lim, 2007), which can lead to cell death. These hypotheses are derived from understanding IDILI from different perspectives or stages, and may not be mutually exclusive. IDILI is likely the result of multiple factors. Most of these hypotheses involve the immune system of the body, which plays a key role in the occurrence and development of IDILI.

**FIGURE 5:**
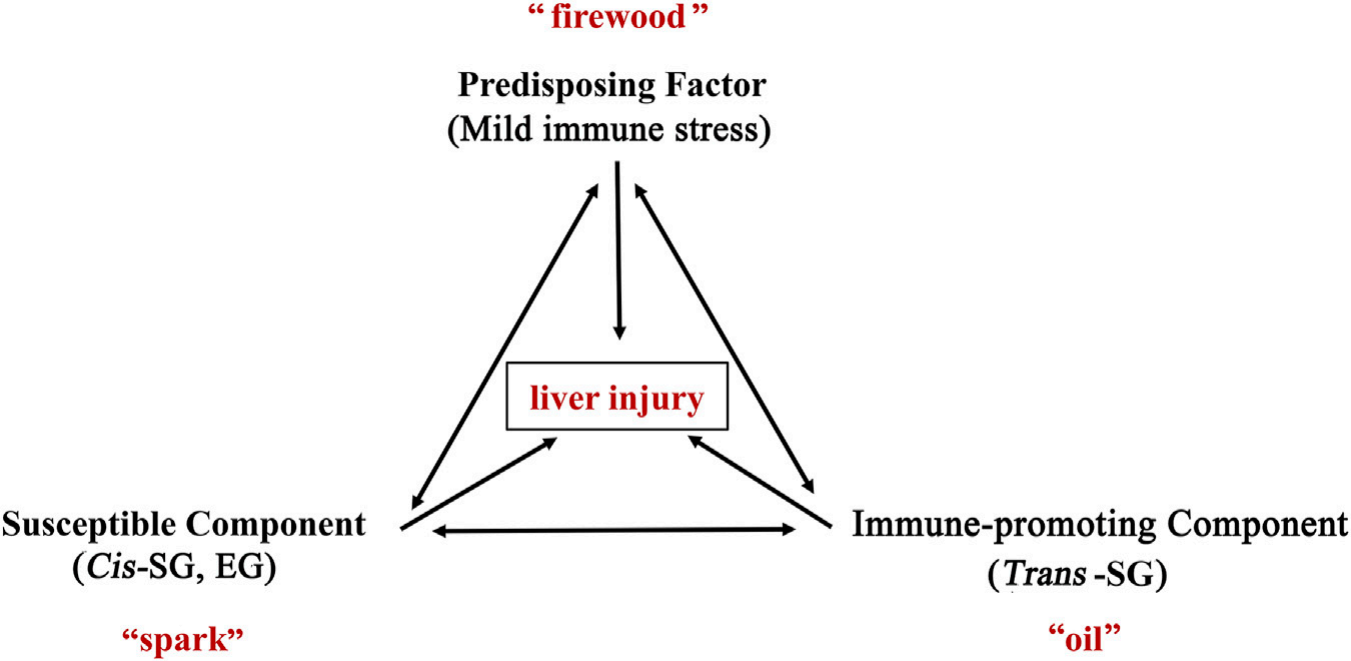
The immunological stress-mediated tri-element injury hypothesis of idiosyncratic toxicity of PM. During an abnormally activated state (mild immune stress) of the body’s immune system, the immune-promoting components in PM (*trans*-SG) may enhance the body’s immune activation, and at the same time increase the susceptibility of the liver to certain components (*cis*-SG, EG), resulting in the overexpression of inflammatory cytokines, leading to liver injury. It is also called the "firewood-oil-spark hypothesis", in which the abnormally activated immune system is equivalent to "firewood", the immune-promoting components in PM are equivalent to "oil", and the potentially susceptible components for liver injury is equivalent to "spark". A single risk factor may not catch fire, but when three risk factors are present at the same time, a small spark may cause a raging fire (acute liver injury). Abbreviations: *trans*-SG, *trans*-2,3,5,4'-tetrahydroxystilbene-2-O-β-glucoside; *cis*-SG, *cis*- 2,3,5,4'-tetrahydroxystilbene-2-O-β-glucoside; EG, emodin-8-O-glucoside; PM, *Reynoutria multiflora* (Thunb.) Moldenke.

Based on the inflammatory stress hypothesis, Rao et al. (Rao et al., 2020) considered PM-HILI to be an immune-mediated idiosyncratic liver injury. Further research has found that PM is more likely to cause liver injury in patients with immune activation. Also, individual components in PM have a potential role in promoting immune activation and causing liver injury. Wang et al. (Wang JB. et al., 2016; Wang J. B. et al., 2016) proposed the immune stress-mediated tri-element injury hypothesis of idiosyncratic toxicity of PM (Figure 5). It states that during an abnormally activated state (MIS) of the body’s immune system, the immune-promoting components in PM (*trans*-SG) may enhance the body’s immune activation, and at the same time increase the susceptibility of the liver to certain components (*cis*-SG, EG), resulting in the overexpression of inflammatory cytokines, leading to liver injury. It is also called the "firewood-oil-spark hypothesis", in which the abnormally activated immune system is equivalent to "firewood", the immune-promoting components in PM are equivalent to "oil", and the potentially susceptible components for liver injury is equivalent to "spark". A single risk factor may not catch fire, but when three risk factors are present at the same time, a small spark may cause a raging fire (acute liver injury). This hypothesis has been experimentally confirmed with animal models, providing new ideas for understanding PM-HILI. Indeed, this hypothesis systematically reveals the characteristics of the multi-component, multi-target effect, and immune synergy of the idiosyncratic HILI, providing new strategies for studying the mechanism of idiosyncratic HILI. In addition, it also suggests that PM should be avoided in combination with immune-promoting herbs and encouraged in combination with immune-suppressing herbs, which may be conducive to reduce liver injury. HLA-B*35:01 allele and rs1055348 may also be potentially involved in PM-HILI, but evidence on the experimental animal models is not yet available and needs to be explored.

## Perspectives

PM-HILI is a typical representative of HILI. Studying the risk factors, hepatotoxic components, and molecular mechanisms of PM-HILI is essential for a thorough understanding of the pathogenesis, treatment, and prevention of HILI. PM-HILI is considered to be IDILI and occurs only in a small number of susceptible individuals. The risk factors of PM-HILI mainly include MIS and gene polymorphism. The specific components responsible for the idiosyncratic hepatotoxicity are mainly *cis*-SG and EG. The processing technology of PM should be improved for quality control of *cis*-SG (<0.10%) and EG (<0.17%). Exposure to light should be avoided during the preparation and storage of PM for preventing the transformation from *trans*-SG to *cis*-SG. Underlying diseases, immune status, genetic background, etc. should be considered comprehensively when using PM and related preparations in clinical practice. The usage of biomarkers, such as HLA-B*35:01 allele, inflammatory cytokines like TNF-α, MCP-1, VEGF, etc. to accurately identify the susceptible populations of PM-HILI is necessary for safe medication. PM should be encouraged in combination with immune-suppressing herbs for preventing liver injury. New mechanisms of PM-HILI need to be found and validated by *in vivo* and *in vitro* studies and complete the current hypothesis in the future.
